# Nomogram predicts CR-POPF in open central pancreatectomy patients with benign or low-grade malignant pancreatic neoplasms

**DOI:** 10.3389/fonc.2022.1030080

**Published:** 2022-12-15

**Authors:** Liu Ouyang, Ren-dong Liu, Yi-wei Ren, Gang Nie, Tian-lin He, Gang Li, Ying-qi Zhou, Zhi-ping Huang, Yi-jie Zhang, Xian-gui Hu, Gang Jin

**Affiliations:** ^1^ Department of the Hepatobiliary and Pancreatic (HBP) Surgery, Changhai Hospital, Naval Medical University, Shanghai, China; ^2^ Department of Hepatobiliary Surgery, General Hospital of Southern Theatre Command, Guangzhou, China

**Keywords:** body mass index (BMI), obesity, pancreatic anastomosis technique, clinically relevant postoperative pancreatic fistula (CR-POPF), central pancreatectomy (CP), nomogram

## Abstract

**Introduction:**

Central pancreatectomy (CP) is a standard surgical procedure for benign and low-grade malignant pancreatic neoplasms in the body and neck of the pancreas. Higher incidence of clinically relevant postoperative pancreatic fistula (CR-POPF) after CP than after pancreaticoduodenectomy (PD) or distal pancreatectomy (DP) has been reported, but no nomogram for prediction of CR-POPF after open CP has been previously established.

**Methods:**

Patients undergoing open CP for benign or low-grade malignant pancreatic neoplasms in the department of Hepatobiliary and Pancreatic (HBP) surgery of Shanghai Changhai Hospital affiliated to Naval Medical University between January 01, 2009 and December 31,2020 were enrolled. Pre-, intra- and post-operative parameters were analyzed retrospectively.

**Results:**

A total of 194 patients, including 60 men and 134 women, were enrolled with median age of 52 years (21~85 years). 84 patients (43.3%) were overweight (BMI>23.0 Kg/m2) and 14 (7.2%) were obese (BMI>28.0 Kg/m2). Pathological diagnoses ranged from serous cystic neoplasm (32.5%), solid pseudopapillary neoplasm (22.2%), pancreatic neuroendocrine tumor (20.1%), intraductal papillary mucinous neoplasm (18.0%) to mucinous cystic neoplasm (5.2%). All patients had soft pancreatic texture. Main pancreatic duct diameters were ≤0.3cm for 158 patients (81.4%) and were ≥0.5cm in only 12 patients (6.2%). A stapler (57.7%) or hand-sewn closure (42.3%) were used to close the pancreatic remnant. The pancreatic anastomosis techniques used were duct to mucosa pancreaticojejunostomy (PJ)-interrupted suture (47.4%), duct to mucosa PJ-continuous suture (43.3%), duct to mucosa “HO” half-purse binding PJ (5.2%) and invaginating pancreaticogastrostomy (4.1%). Post-surgical incidences of CR-POPF of 45.9%, surgical site infection of 28.9%, postpancreatectomy hemorrhage of 7.7% and delayed gastric emptying of 2.1% were found. Obesity and pancreatic anastomosis technique were independent risk factors of CR-POPF, with a concordance index of 0.675 and an Area Under the Curve of 0.678.

**Discussion:**

This novel nomogram constructed according to obesity and pancreatic anastomosis technique showed moderate predictive performance of CR-POPF after open CP.

## Introduction

Many benign or low-grade malignant pancreatic neoplasms are asymptomatic but recent advances in imaging techniques have allowed increased detection rates (1). Prognoses tend to be good and when complete resection (R0 resection) is required, parenchyma-sparing surgeries better preserve the exocrine and endocrine pancreatic function (2). Parenchyma-sparing surgeries include central pancreatectomy (CP), duodenum-sparing head resection and enucleation. CP preserves much of the pancreatic head and distal pancreatic volume, allowing better retention of exocrine and endocrine function than pancreaticoduodenectomy (PD) or distal pancreatectomy (DP) and has become the standard surgical procedure for benign and low-grade malignant pancreatic neoplasms in the body and neck of the pancreas (3–6).

However, CP leaves two divided pancreatic remnants, creating more opportunities for postoperative pancreatic fistula (POPF) formation. There have been many reports of higher POPF incidence following CP than after PD or DP (5–10). POPF is a severe and challenging complication of CP, contributing to post-operative morbidity, due to post-pancreatectomy hemorrhage, surgical site infection or abdominal abscess, mortality and prolonged hospitalization (5–10). It has been described as the “Achilles heel” of CP (11). Establishment of a system predictive of CR-POPF risk after CP is necessary to inform individualized treatment plans for affected patients. However, since CP is seldom performed (8), post-CP CR-POPF prediction has not been developed. Nomograms have been widely used to predict CR-POPF risk after pancreatectomy with favorable results (12–14). The current study uses data from the largest single-center open CP cohort so far reported to establish a nomogram predictive of CR-POPF for post-CP patients with benign and low-grade malignant pancreatic neoplasms.

## Patients and methods

### Patients and study design

**Figure 1:**
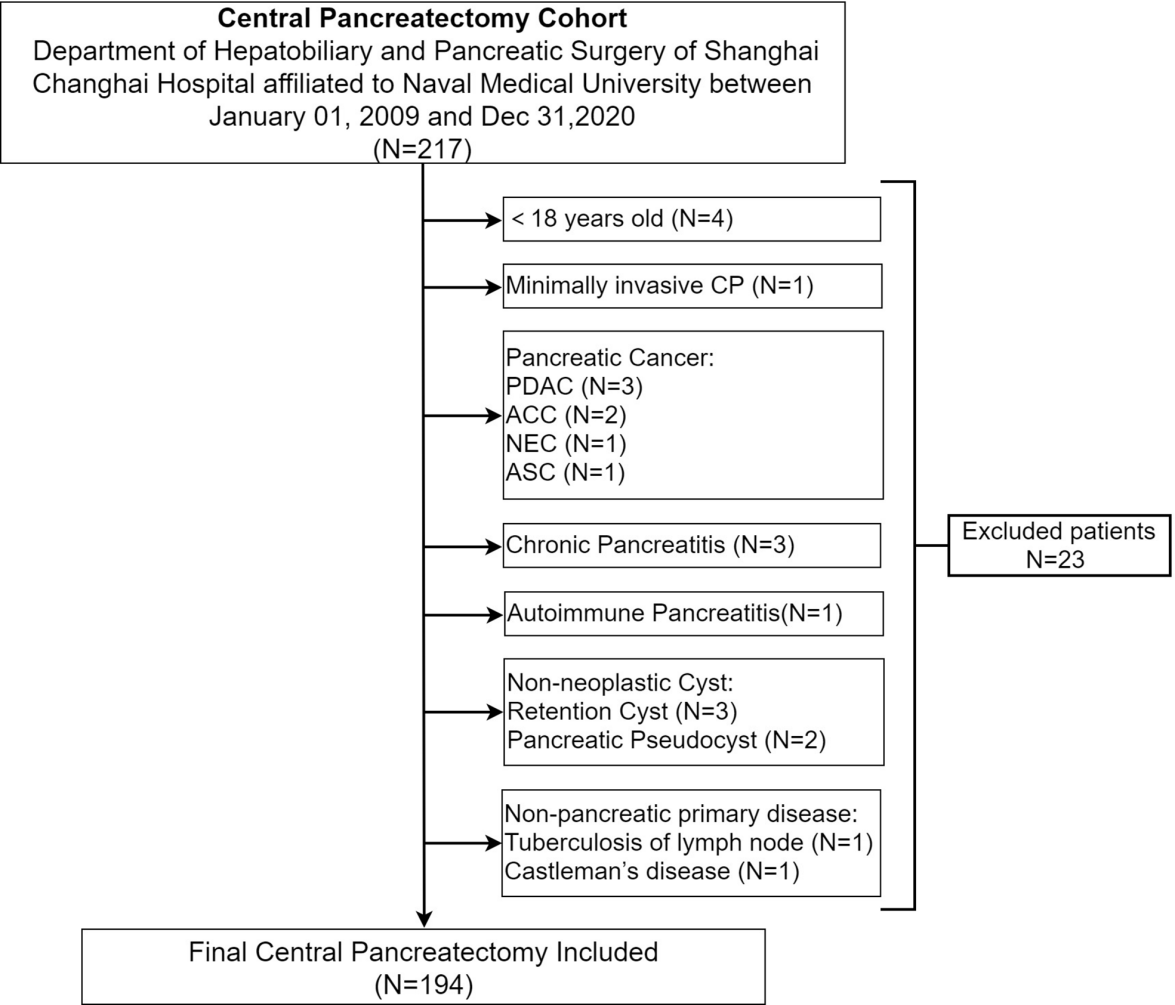
Flow chart of open central pancreatectomy selection.

A total of 194 patients undergoing CP for benign and low-grade malignant pancreatic neoplasms in the department of Hepatobiliary and Pancreatic (HBP) surgery of Shanghai Changhai Hospital affiliated to Naval Medical University between January 01, 2009 and December 31,2020 were enrolled. Inclusion criteria were as follows: 1. >18 years; 2. benign and low-grade malignant pancreatic neoplasm; 3. open central pancreatectomy (OCP). Exclusion criteria were as follows: 1. minimally invasive central pancreatectomy (MICP), including laparoscopic or robotic CP; 2. pancreatic cancer, including pancreatic ductal adenocarcinoma (PDAC) and its subtypes, adenosquamous carcinoma (ASC), acinar cell carcinoma (ACC) and neuroendocrine carcinoma (NEC); 3. chronic pancreatitis; 4. non-pancreatic primary disease, tuberculosis of lymph nodes, Castleman’s disease. (**Figure 1**).

Comprehensive demographic, pre-, intra- and post- operative data of all patients who underwent CP in the 12 years of interest were collated from electronic medical records. Perioperative parameters were analyzed retrospectively. All patients gave written informed consent for participation, and the Ethics Committee of Shanghai Changhai Hospital granted the ethical approval.

### Open central pancreatectomy surgical techniques

Midline upper abdominal laparotomy was performed and the pancreas was exposed by division of the gastrocolic ligament. The inferior border of the pancreas was mobilized in front of superior mesenteric vein (SMV) and a tunnel created between the posterior surface of the pancreatic neck and portal vein (PV)/SMV. If necessary, resection was extended to the right of the PV/SMV with division of the gastroduodenal artery (GDA) and pancreatic head transection along the left of bile duct.

**Figure 2:**
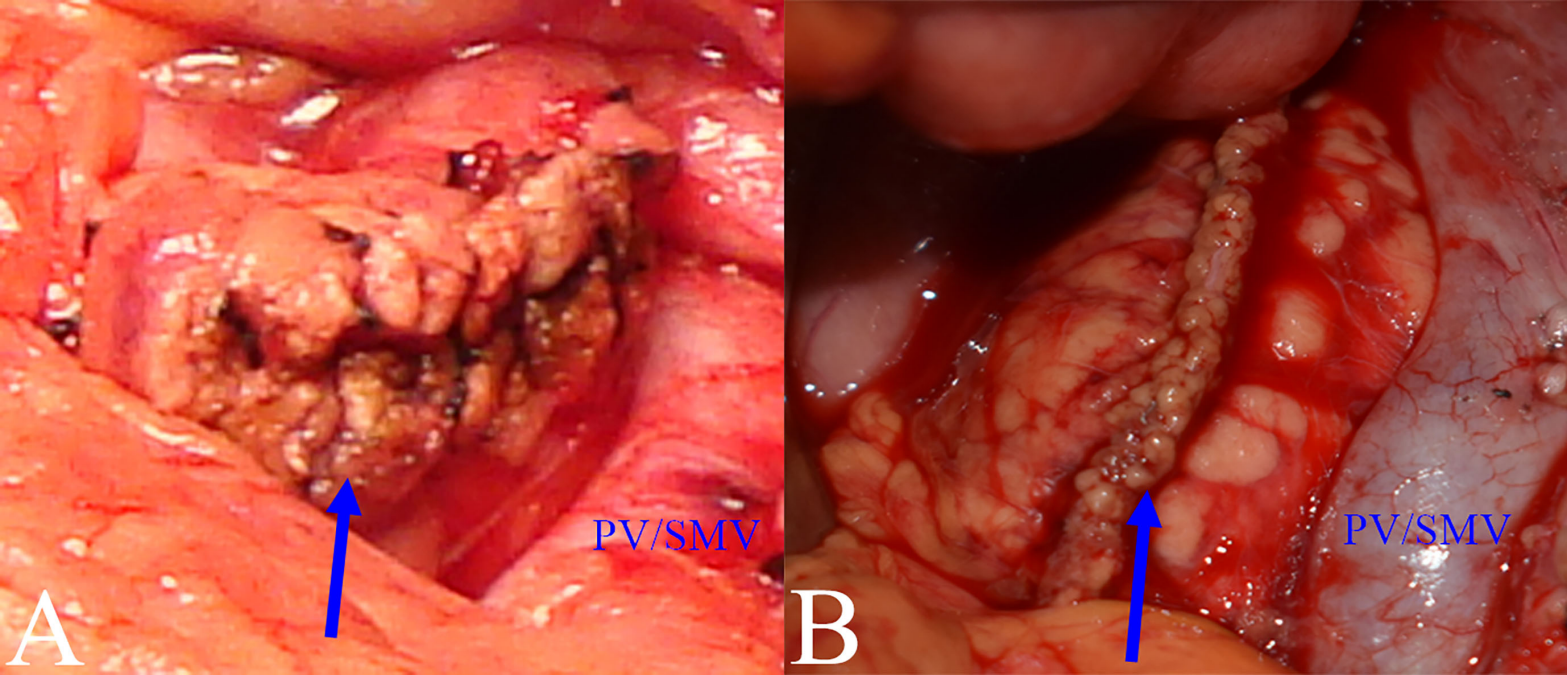
Closure methods for pancreatic remnants. **(A)** Hand-sewn closure: pancreatic remnant after closure (blue arrow). **(B)** Stapler closure: pancreatic remnant after closure (blue arrow).

**Figure 3:**
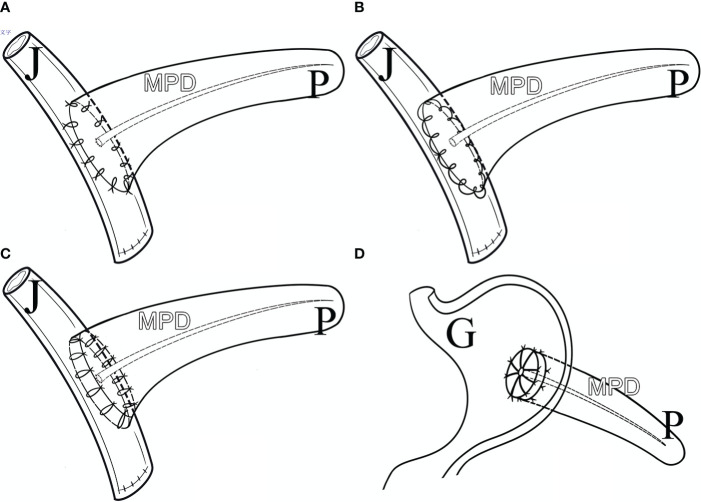
Pancreatic Anastomosis Techniques. **(A)** Duct to Mucosa PJ (Interrupted Suture). **(B)** Duct to Mucosa PJ (Continuous Suture). **(C)** Duct to Mucosa “HO” half-purse binding PJ. **(D)** Invaginating PG. P, pancreas; J, jejunum; G, gastro; MPD, main pancreatic duct.

The proximal pancreatic remnant was closed by hand-sewn closure or stapler. Use of suture involved location of the main pancreatic duct (MPD), which was not usually dilated in the transection, and oversewing of the MPD with a silk thread before closure of the proximal pancreatic stump by vertical mattress suture with silk thread. Use of stapler involved transection of the pancreatic neck by mechanical stapler (**Figure 2**). After the pancreatic neck was transected, by taking care to ligate and cut small branches to or from the pancreas, the PV/SMV, splenic veins (SV) and splenic artery (SA) were freed from the posterior of the pancreatic body, SV and SA were carefully protected during this process. A lesion in the body or neck of the pancreas was excised with a margin of 1 to 2 mm from both pancreatic stumps. Approximately 1 cm distal to the pancreatic stumps was mobilized. An appropriately sized stent was placed within the MPD. Different pancreatic anastomosis techniques (**Figure 3**) were performed in the distal pancreatic remnant: end-to-side duct to mucosa pancreaticojejunostomy (PJ) (interrupted suture and continuous suture), end-to-side duct to mucosa “HO” half-purse binding PJ or invaginating pancreaticogastrostomy (PG). PJ involved two-layer and duct-to-mucosa anastomosis, with continuous suture (15), interrupted suture, or “HO” half-purse binding PJ (16). The pancreatic stump was closed directly by the jejunal wall with linear MPD drainage. Invaginating PG involved two-layer invaginating anastomosis with interrupted suture. Two silicone or latex suction drains were placed under the pancreatic remnant and the PJ/PG anastomosis after CP and pulled out through the left and/or right of the midline upper abdominal incision.

### Post-operative management

Patients received 24h antibiotic prophylaxis following open CP. Octreotide was only given as a POPF prophylactic in cases where there was evidence of CR-POPF or acute pancreatitis. Laboratory testing of drain fluid amylase activity was conducted routinely every 2 days from postoperative day (POD) 1 until drainage removal. Abdominal drainage volume was measured every day. When the drain amylase level less than 5000 U/L and drain output less than 300 mL per day, and there were no signs of intraabdominal infection, we removed the abdominal drain. An emergency CT was performed if signs of intraabdominal infection were detected and if fluid accumulation could be seen, a percutaneous drainage tube was placed using ultrasound or CT guidance.

### Definition of post-operative complications

Grade B and grade C, but not grade A, POPFs were considered to have clinical significance and to be defined as CR-POPF, based on the definition given by the International Study Group for Pancreatic Fistula (ISGPF) in 2016 (17). Post-pancreatectomy hemorrhage (PPH) was diagnosed and graded based on the definition given by the International Study Group of Pancreatic Surgery (ISGPS): PPH-ISGPS (2007) (18), Delayed Gastric Emptying (DGE) was diagnosed and graded based on the ISGPS definition: DGE-ISGPS (2007) (19). Post-operative complications were assessed based on the Clavien-Dindo classification (2004) (20).

### Statistical analysis

Continuous variables are described as medians (quadrants) and are compared using the Mann-Whitney U test. Categorical variables are reported as integers and percentages and are compared by the *χ2* test or Fisher’s exact test. Cut-off values for certain parameters were determined by receiver operating characteristic (ROC) curve. Univariate and multivariate risk factor analysis was conducted by using logistic regression analysis, and a nomogram predicts CR-POPF was established based on the result of multivariate risk factor analysis. Three statistical approaches were used to verify the nomogram: concordance index analysis, ROC curve and calibration plot. Statistical analyses were conducted using GraphPad Prism 9, SPSS 24.0 software and R software version 4.1.2. All statistical significance levels were two-sided and a value of *p* < 0.05 was considered to indicate significance.

## Results

### Baseline features

The 194 patients consisted of 60 men and 134 women, with a median age of 52 years (range: 21~85 years), among them 148 patients>50 years and 46 patients ≤ 50 years. 113 patients (58.2%) were asymptomatic, 70 (36.1%) complained of abdominal discomfort, 9 (4.6%) had a history of hypoglycemia prior to surgery and 2 (1.0%) had experienced weight loss. 16 patients (8.2%) had history of diabetes and 14 (7.2%) had history of pancreatitis. 84 patients (43.3%) were overweight (BMI>23.0 Kg/m^2^) and 14 (7.2%) were obese (BMI>28.0 Kg/m^2^). Most patients had normal CA19-9 and CEA levels.

The most common pathological diagnosis was serous cystic neoplasm (63/194, 32.5%), followed by solid pseudopapillary neoplasm (43/194, 22.2%), pancreatic neuroendocrine tumor (39/194, 20.1%), intraductal papillary mucinous neoplasm (35/194, 18.0%), mucinous cystic neoplasm (10/194, 5.2%) and other tumors (4/194, 2.1%). The mean duration of surgery was 128min (range: 60~275 min) and the median estimated intraoperative blood loss was 179 ml (range: 20~800 ml) with 3 patients receiving intraoperative blood transfusion. A soft pancreatic texture was present in all cases. The MPD diameter was ≤0.3cm in 158 patients with only 12 patients having an MPD > 0.5cm in diameter.

The pancreatic remnant was closed by either stapler (112/194, 57.7%) or hand-sewn closure (82/194, 42.3%). Four pancreatic anastomosis techniques were used (**Table 1**): duct to mucosa PJ (interrupted suture) (92/194, 47.4%), duct to mucosa PJ (continuous suture) (84/194, 43.3%), duct to mucosa “HO” half-purse binding PJ (10/194, 5.2%) and invaginating PG (8/194, 4.1%).

**Table 1:** Clinicopathological Characteristics and Postoperative Complications.

Variable	CP (N=194)	CR-POPF		P-value	Severe Complication		P-value
		Absent (N=93) (50.1%)	Present (N=79) (45.9%)		Absent (N=124) (64.2%)	Present (N=69) (35.8%)	
Gender				0.059			0.072
Male	60 (30.9%)	25 (43.9%)	32 (56.1%)		33 (55.0%)	27 (45.0%)	
Female	134 (69.1%)	68 (59.1%)	47 (40.9%)		91 (68.4%)	42 (31.6%)	
Age (year)				0.135			0.165
>50	148 (76.3%)	76 (57.1%)	57 (42.9%)		99 (66.9%)	49 (33.1%)	
≤50	46 (23.7%)	17 (43.6%)	22 (56.4%)		25 (55.6%)	20 (44.4%)	
Symptoms				0.102			0.088
Present	81 (41.8%)	42 (61.8%)	26 (38.2%)		57 (71.3%)	23 (28.7%)	
Absent	113 (58.2%)	51 (49.0%)	53 (51.0%)		67 (59.3%)	46 (40.7%)	
History of Diabetes				0.085			0.138
Present	16 (8.2%)	10 (76.9%)	3 (23.1%)		13 (81.3)	3 (18.7%)	
Absent	178 (91.8%)	83 (52.2%)	76 (47.8%)		111 (62.7%)	66 (37.3%)	
History of Hypertension				0.341			0.220
Present	41 (21.1%)	19 (47.5%)	21 (52.5%)		23 (56.1%)	18 (43.9%)	
Absent	153 (78.9%)	74 (56.1%)	58 (43.9%)		101 (66.4%)	51 (33.6%)	
History of Pancreatitis				0.056			0.246
Present	14 (7.2%)	9 (81.8%)	2 (18.2%)		11 (78.6%)	3 (21.4%)	
Absent	180 (92.3%)	84 (52.2%)	77 (47.8%)		113 (63.1%)	66 (36.9%)	
History of Abdominal Surgery				0.436			0.392
Present	50 (25.8%)	20 (48.8%)	21 (51.2%)		29 (59.2%)	20 (40.8%)	
Absent	144 (74.2%)	73 (55.7%)	58 (44.3%)		95 (66.0%)	49 (34.0%)	
History of Smoking				0.526			0.467
Present	21 (10.8%)	11 (61.1%)	7 (38.9%)		15 (71.4%)	6 (28.6%)	
Absent	173 (89.2%)	82 (53.2%)	72 (46.8%)		109 (63.4%)	63 (36.6%)	
History of Alcoholism				0.436			0.697
Present	10 (5.2%)	6 (66.7%)	3 (33.3%)		7 (70.0%)	3 (30.3%)	
Absent	184 (94.8%)	87 (53.4%)	76 (46.6%)		117 (63.9%)	66 (36.1%)	
Overweight				0.060			0.026
BMI>23.0 Kg/m2	84 (43.3%)	35 (46.1%)	41 (53.9%)		46 (55.4%)	37 (44.6%)	
BMI ≤ 23.0 Kg/m2	110 (56.7%)	58 (60.4%)	38 (39.6%)		78 (70.9%)	32 (29.1%)	
Obesity				0.004			0.045
BMI>28.0 Kg/m2	14 (7.2%)	2 (15.4%)	11 (84.6%)		5 (38.5%)	8 (61.5%)	
BMI ≤ 28.0 Kg/m2	180 (92.8%)	91 (57.2%)	68 (42.8%)		119 (66.1%)	61 (33.9%)	
ASA score				0.264			0.820
1	104 (53.6%)	47 (52.2%)	43 (47.8%)		69 (67.0%)	34 (33.0%)	
2	60 (30.9%)	31 (56.4%)	24 (43.6%)		36 (60.0%)	24 (40.0%)	
3	30 (15.5%)	15 (55.6%)	12 (44.4%)		19 (63.3%)	11 (36.7%)	
Pre-operative parameter							
CA199	24.76 ± 82.94	14.36 ± 14.92	19.84 ± 48.01	0.024	24.92 ± 94.03	24.47 ± 58.16	0.840
CEA	2.19 ± 2.02	2.35 ± 2.56	1.95 ± 1.31	0.122	2.31 ± 2.32	1.97 ± 1.32	0.180
Total bilirubin	12.26 ± 5.01	12.14 ± 4.7	12.87 ± 5.63	0.503	11.96 ± 4.55	12.81 ± 5.77	0.137
Albumin	42.10 ± 3.77	42.19 ± 3.57	42.6 ± 3.85	0.416	41.99 ± 3.67	42.31 ± 4	0.446
Blood glucose	5.35 ± 1.75	5.36 ± 1.28	5.26 ± 1.77	0.686	5.45 ± 1.76	5.18 ± 1.74	0.570
Serum amylase	71.33 ± 44.03	69.03 ± 40.42	72.47 ± 53.22	0.590	75.08 ± 50.04	62.33 ± 26.06	0.186
PT, s	12.95 ± 0.76	12.96 ± 0.84	12.95 ± 0.69	0.259	12.97 ± 0.82	12.9 ± 0.65	0.131
WBC, 10^9^/L	5.72 ± 1.66	5.51 ± 1.71	5.91 ± 1.59	0.582	5.61 ± 1.72	5.9 ± 1.56	0.340
HGB, g/L	131.93 ± 14.64	131.41 ± 14.16	134.64 ± 14.87	0.489	130.89 ± 14.23	133.82 ± 15.4	0.211
PLT	210.15 ± 53.93	210.2 ± 52.67	212.64 ± 56.33	0.619	209.8 ± 54.68	210.78 ± 53.4	0.755
CRP	3.74 ± 10.00	5.02 ± 14.88	2.66 ± 1.93	0.095	4.66 ± 13.21	2.44 ± 1.21	0.118
PCT	0.03 ± 0.03	0.02 ± 0.01	0.04 ± 0.04	0.206	0.03 ± 0.01	0.04 ± 0.04	0.115
Intra- and post-operative parameter							
Closure methods of pancreatic remnant				0.207			0.055
Stapler	112 (57.7%)	50 (50.0%)	50 (50.0%)		65 (58.6%)	46 (41.4%)	
Hand-sewn closure	82 (42.3%)	43 (59.7%)	29 (40.3%)		59 (72.0%)	23 (28.0%)	
Pancreatic anastomosis technique				0.004			<0.001
Duct to Mucosa PJ (Interrupted Suture)	92 (47.4%)	51 (68.0%)	24 (32.0%)		72 (78.3%)	20 (21.7%)	
Duct to Mucosa PJ (Continuous Suture)	84 (43.3%)	33 (41.8%)	46 (58.2%)		43 (51.8%)	40 (48.2%)	
Duct to Mucosa “HO” half-purse binding PJ	10 (5.2%)	3 (30.0%)	7 (70.0%)		3 (30.0%)	7 (70.0%)	
Invaginating PG	8 (4.1%)	6 (75.0%)	2 (25.0%)		6 (75.0%)	2 (25.0%)	
Pathology				0.820			0.954
SCN	63 (32.5%)	29 (49.2%)	30 (50.8%)		40 (63.5%)	23 (36.5%)	
SPN	43 (22.2%)	21 (55.3%)	17 (44.7%)		28 (65.1%)	15 (34.9%)	
NET	39 (20.1%)	19 (54.3%)	16 (45.7%)		27 (69.2%)	12 (30.8%)	
IPMN	35 (18.0%)	18 (56.3%)	14 (43.7%)		21 (60.0%)	14 (40.0%)	
MCN	10 (5.2%)	4 (80.0%)	1 (20.0%)		6 (66.7%)	3 (33.3%)	
Others (Paraganglioma, PEComa)	4 (2.1%)	2 (66.7%)	1 (33.3%)		2 (50.0%)	2 (50.0%)	
Largest tumor diameter, cm				0.094			0.095
≥2.0	154 (79.4%)	77 (57.5%)	57 (42.5%)		101 (67.3%)	49 (32.7%)	
<2.0	40 (20.6%)	16 (42.1%)	22 (57.9%)		23 (53.5%)	20 (46.5%)	
Diameter of main Pancreatic Duct, cm				0.777			0.737
≤0.1	22 (11.3%)	9 (53.0%)	8 (47.0%)		16 (72.7%)	6 (27.3%)	
0.2	59 (30.4%)	28 (53.8%)	24 (46.2%)		40 (69.0%)	18 (31.0%)	
0.3	77 (39.7%)	40 (54.1%)	34 (45.9%)		43 (55.8%)	34 (44.2%)	
0.4	24 (12.4%)	10 (47.6%)	11 (52.4%)		15 (62.5%)	9 (37.5%)	
≥0.5	12 (6.2%)	6 (75.0%)	2 (25.0%)		10 (83.3%)	2 (16.7%)	
Operation Time, min	128.09 ± 30.95	128.17 ± 34.46	128.86 ± 27.66	0.187	128.91 ± 33.05	126.67 ± 27.45	0.194
Estimated Blood Loss, ml	179.23 ± 113.66	167.96 ± 107.44	197.47 ± 128.33	0.732	173.15 ± 103.02	191.3 ± 131.72	0.250
Post-operative PH	7.37 ± 0.06	7.38 ± 0.05	7.37 ± 0.06	0.498	7.38 ± 0.05	7.37 ± 0.06	0.397
Post-operative BE	-2.11 ± 2.06	-2.06 ± 1.97	-2.1 ± 2.15	0.538	-2.2 ± 2	-1.97 ± 2.21	0.609

### Post-operative complications

Among the 194 patients enrolled in this study, we have not found the amylase activity of fluid output *via* the operatively placed drain in 22 of them, so we can’t judge whether they have POPF or not, in the remaining 172 patients, the incidences of Grades B and C POPF were 45.3% (78/172) and 0.58% (1/172), respectively. 12 patients (6.2%) received postoperative erythrocyte transfusion. 56 patients (28.9%) had surgical site infection (SSI). 15 patients (7.7%) had post-pancreatectomy hemorrhage (PPH): 3 (20.0%) experienced early and 12 (80.0%) experienced late hemorrhage; 11 (73.3%) had extraluminal and 4 (26.7%) had intraluminal hemorrhage; 2 cases had grade A, 10 grade B and 3 grade C PPH. 4 patients (2.1%) had postoperative delayed gastric emptying (DGE): 2 cases were grade A and 2 were grade C. 1 patient (0.52%) died on POD 3 and the cause of death is diabetic ketoacidosis with septic shock. The average length of the hospital stay is 9.8 days (range: 3~47 d).

The rate of postoperative diabetes mellitus was 5.2% (10/194) and of postoperative pancreatitis, 5.7% (11/194). 1 patient with a pathological diagnosis of pancreatic neuroendocrine tumor experienced symptomatic PJ stricture 6 years after CP which was treated by resection of the PJ, followed by new two-layer end-to-side PJ with internal drainage of the Wirsung duct. 1 patient with a pathological diagnosis of intraductal papillary mucinous neoplasm relapsed 18 months after CP with pancreatic ductal adenocarcinoma and was treated by pylorus preserving pancreaticoduodenectomy (PPPD).

### Univariate and multivariate analysis of CR-POPF-associated factors

The following perioperative parameters were conducted to univariate and multivariate analyses: gender, age, symptoms, hypertension, diabetes, pancreatitis, abdominal surgery, smoking, alcoholism, BMI, ASA score, closure methods of pancreatic remnant, pancreatic anastomosis technique, pathology, largest tumor diameter, diameter of pancreatic duct, operation time, estimated blood loss and laboratory tests of blood or serum. The results showed that BMI ≥28kg/m2 (hazard ratio [HR] 7.663; 95% confidence interval [CI] 1.557–37.721) and pancreatic anastomosis technique (invaginating PG was set as the reference: hazard ratio for duct to mucosa PJ-Continuous suture: 4.364 [0.701, 27.183]; duct to mucosa “HO” half-purse binding PJ: 7.328 [0.778, 69.001]; duct to mucosa PJ-Interrupted suture: 1.489 [0.237, 9.348]) were independent risk factors for CR-POPF (**Table 2**).

**Table 2:** Univariate and mutivariate factor analysis for POPF.

	Univariate				Mulitivariate			
Variable	P	HR	Lower limit	Upper limit	P	HR	Lower limit	Upper limit
Gender, male:female	0.060	1.852	0.975	3.519				
Symptoms, present:absent	0.103	0.596	0.320	1.110				
History of Diabetes, present:absent	0.099	0.328	0.087	1.235				
History of Pancreatitis, present:absent	0.076	0.242	0.051	1.157				
BMI, ≥28: <28kg/m^2^	0.011	7.360	1.579	34.300	0.012	7.663	1.557	37.721
ASA score	0.669	0.915	0.609	1.375				
CA19-9, ≥37: <37u/ml	0.548	1.515	0.391	5.871				
Closure methods of pancreatic remnant, staple:hand suture	0.208	1.483	0.803	2.736				
Largest tumor diameter, ≥2: <2cm	0.127	0.564	0.270	1.176				
Diameter of Pancreatic Duct, ≥2: <2mm	0.923	0.970	0.526	1.789				
Albumin(preoperative minus POD1), ≥0: <0	0.127	0.480	0.187	1.231				
Pathology, SCN : SPN:NET : IPMN : MCN:Others	0.926	NA	NA	NA				
Pancreatic anastomosis technique	0.010				0.012			
Duct to Mucosa PJ (Continuous Suture)	0.104	5.833	0.696	48.873	0.114	4.364	0.701	27.183
Duct to Mucosa “HO” half-purse binding PJ	0.852	1.176	0.213	6.505	0.082	7.328	0.778	69.001
Duct to Mucosa PJ (Interrupted Suture)	0.150	3.485	0.637	19.070	0.671	1.489	0.237	9.348
Invaginating PG	Ref.	Ref.	Ref.	Ref.	Ref.	Ref.	Ref.	Ref.

*P < 0.05 by Logistic regression model.

Cut-off value of tumour diameter was calculated by ROC curve.

NA, not applicable.

### Construction and validation of nomogram

**Figure 4:**
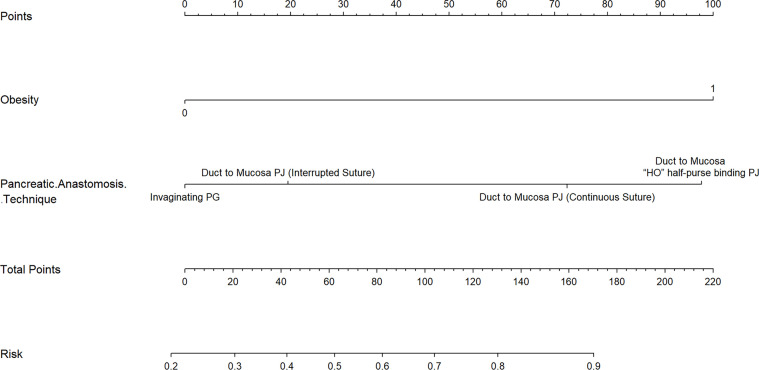
A Novel Nomogram for Predicting CR-POPF in OCP Patients. This nomogram is used by adding the points identified by each variable on the points scale. The sum of these points is projected on the bottom scale and indicates the rate of CR-POPF in individual patients. PJ: pancreaticojejunostomy; PG: pancreaticogastrostomy.

**Figure 5:**
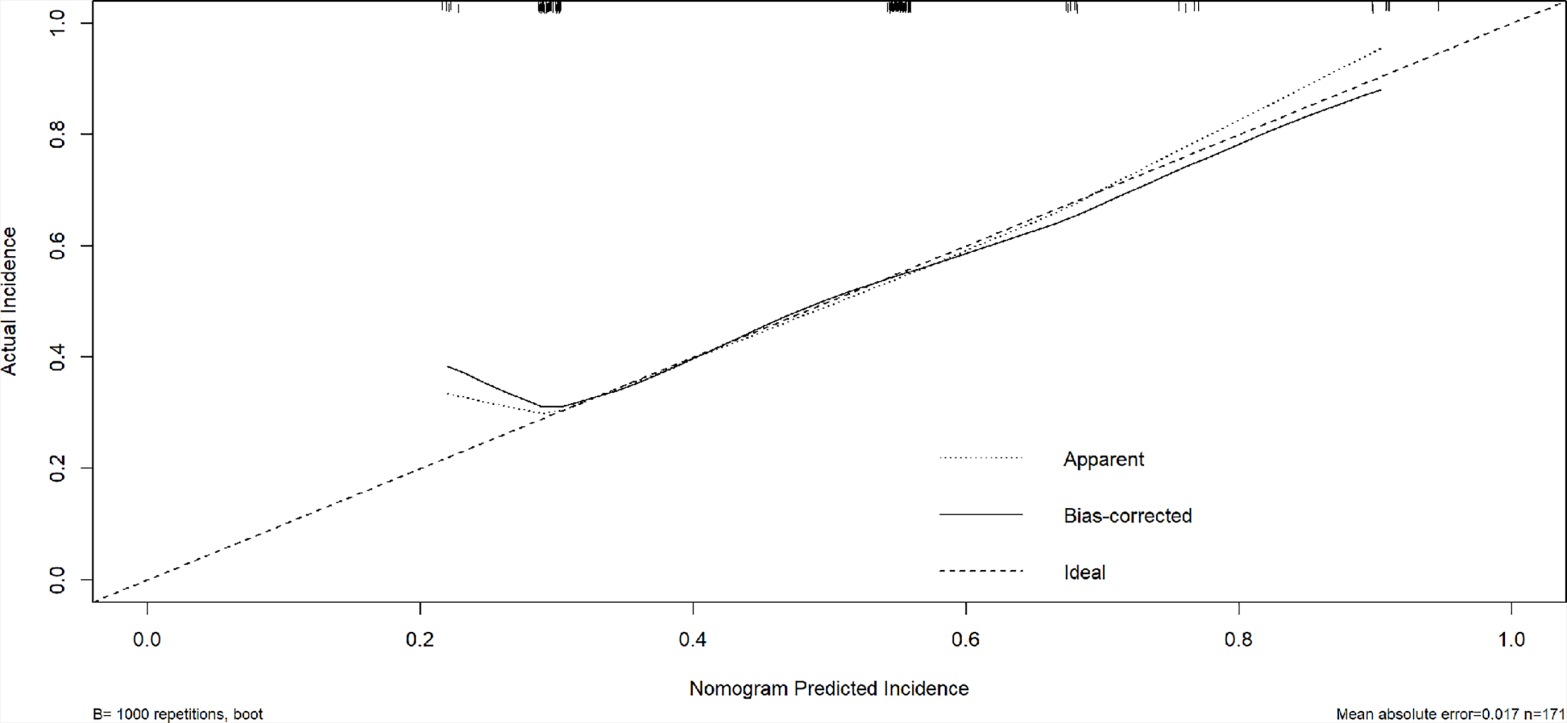
ROC of the Novel Nomogram of CR-POPF in OCP Patients. A ROC curve was constructed to evaluate the model. The area under the ROC curve was 0.678. ROC, receiver operating characteristic.

**Figure 6:**
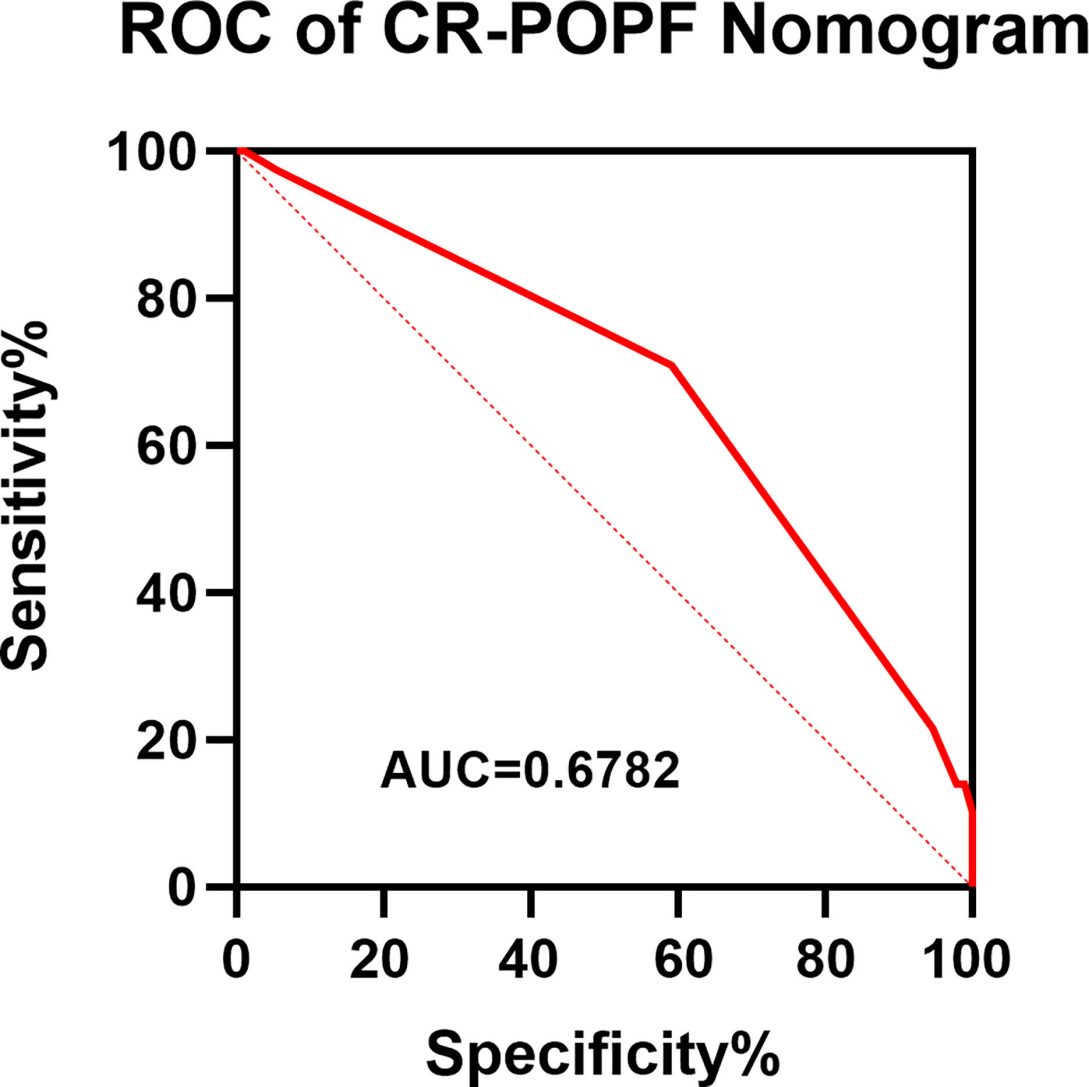
Calibration plot of nomogram predicts CR-POPF. The X–axis represents the nomogram–predicted survival, while the actual survival was plotted on the Y–axis. And the apparent incidence of CR-POPF, the ideal incidence, the bias-corrected incidence were shown as different lines.

Obesity (BMI≥28kg/m2) and pancreatic anastomosis technique (**Table 2**) were selected for construction of the nomogram for prediction of CR-POPF (**Figure 4**) and validation was performed by concordance test and ROC curve (**Figure 5**). The concordance test produces results that range from 0.5, indicating a totally random predictive performance, to 1.0, indicating a perfect predictive performance. A moderate predictive performance of the current nomogram was demonstrated by a concordance index of 0.675 and an Area Under the ROC Curve of 0.678 (**Figure 5**). The close alignment of the three lines representing apparent incidence, ideal incidence and optimum-corrected should be noted as an indication of satisfactory calibration (**Figure 6**).

## Discussion

The recent advances in imaging techniques have led to the diagnosis of more asymptomatic benign or low-grade malignant pancreatic neoplasms (1). The prognosis of benign or low-grade malignant pancreatic neoplasms is good and parenchyma-preserving surgeries are often used for resection in order to preserve the functions of the pancreas (2). In 1957, the first 2 cases of CP with pancreaticojejunostomy (PJ) were described in 2 patients with chronic pancreatitis (8) and CP was first proposed for treatment of neoplasms in 1984 (8). CP has since become a standard surgical technique for the treatment of benign and low-grade malignant lesions located in the pancreatic body and neck (3–6). The advantages of CP include (21): (1) the endocrine and exocrine function of the pancreas were better preserved; (2) preservation of spleen. The rate of post-operative diabetes mellitus was 5.2% (10/194) in the current study.

However, the two separated edges of the pancreas generated by CP generate increased opportunity for the formation of postoperative pancreatic fistula (POPF). Besides, patients for whom CP is advised usually have benign or low-grade pancreatic malignancies with soft texture and a small MPD in the vast majority of cases, all of these have been demonstrated to be significant risk factors for POPF (12–14). These risk factors are independent of the surgeon’s intervention and the incidence of POPF after CP has been reported to exceed that after standard PD or DP (5–10). POPF remains a severe and challenging complication of CP and is a key contributor to most of the postoperative complications, such as post-pancreatectomy hemorrhage and abdominal abscess, promoting operation-related morbidity, mortality and prolonged hospitalization (5–10). Indeed, POPF is the “Achilles heel” of CP (11). Patients with pancreatic cancer and chronic pancreatitis were excluded in the current study, and 81.4% (158/194) of the cohort had a main pancreatic duct diameter ≤ 0.3cm, the rate of CR-POPF was 45.9%, the incidence of PPH was 7.7%, of SSI 28.9%, of DGE 2.1% and of death 0.52%.

The establishment of a system to predict incidence of CR-POPF after CP is expected to contribute to better patient management. However, the rarity with which CP is performed (8) has meant that such predictions have not been standardized. The largest single-center series of CPs to be published to date are reported during the current study and univariate and multivariate analyses demonstrated that obesity and pancreatic anastomosis technique were independent risk factors of CR-POPF.

Many studies have reported that obesity is an independent risk factor for CR-POPF (22–25). Both the modified Fistula Risk Score (mFRS) (13) and the alternative Fistula Risk Score (aFRS) (14), used to determine clinical risk of pancreatic fistula, incorporate BMI. The prevalence of obesity has tripled since 1975 and continues to show a pandemic-related trend of global increase, according to the World Health Organization (WHO). Surgeons will operate with increasing frequency on overweight and obese people as a consequence. The pancreas, which buried behind the stomach, is a retroperitoneal organ. The enlarged anterior and posterior diameter of the abdominal cavity in obese patients, combined with excessive abdominal and visceral adipose tissue (26, 27), reduces the operative space and may also result in a thickening of the omentum or mesentery. Increased fat deposition in visceral organs and a heavy omentum restrict surgical exposure and blur the operative field, precipitating technical difficulties, especially pancreatic anastomosis (28). Hence, the higher the BMI, the higher the incidence of post-pancreatectomy complications, including POPF, SSI and DGE.

Soft texture of the pancreas is a highly relevant risk factor for CR-POPF (12–14). Patients with a high BMI and increased fatty infiltration have been shown to have a higher rate of soft pancreas (23). In addition, adipose tissue has a poor blood supply and heals slowly, contributing to higher POPF incidence in obese patients.

The common complication of post-operative peripancreatic fluid accumulation in overweight/obese patients may be attributed to a wider resection area, more tissue damage, larger dead space, more frequent drainage dysfunction and delayed mobilization than in patients with normal BMI (29). Obese patients also have a stronger inflammatory response to surgical invasion (30) and high levels of inflammatory factors in patients with high BMI have also been found (31). Accumulation of postoperative peripancreatic fluid and infection may activate pancreatic enzymes, promoting CR-POPF formation (17).

In addition, obesity is associated with comorbidities, such as diabetes mellitus, insulin resistance, chronic inflammation, hypertension, cardiovascular diseases, pulmonary diseases and hormones associated with metabolic syndrome (32), which may also contribute to worse perioperative outcomes, especially CR-POPF.

No statistical differences have been demonstrated between the two closure methods of pancreatic remnant: hand-sewn closure and stapler (33). The incidence of POPF following use of stapler was 50% and following hand sewn suture was 40.3% during the current study, with no statistically significant difference (p=0.207).

Four pancreatic anastomosis techniques were performed during the present study and incidences of CR-POPF from low to high were: invaginating PG (25%), duct to mucosa PJ-interrupted suture (32%), duct to mucosa PJ-continuous suture (58.2%) and duct to mucosa “HO” half-purse binding PJ (70%), with statistically significant differences (p=0.004). 81.4% patients had a main pancreatic duct diameter ≤ 0.3cm, making invaginating PG superior to duct to mucosa PJ (34).

We acknowledge some limitations to this study. First, it is a single center retrospective study, the retrospective nature is subject to selection bias, despite great efforts being made to seek information from medical records. Second, although the sample size was the largest published so far, it was not large enough to divide into separate cohorts for internal validation. Third, skinfold thickness, waist circumference and bioelectrical impedance are alternative measurements of obesity (26) but are expensive, difficult to standardize or not widely available. As a result, BMI was used as a reasonable indicator of body fat in the current article. Fourth, external validation was not performed and multicenter prospective randomized controlled trials are required to verify the findings in the future.

In conclusion, rates of CR-POPF in post-central pancreatectomy patients with benign or low-grade malignant pancreatic neoplasms were high (45.9%). Obesity and pancreatic anastomosis technique are two independent risk factors of CR-POPF. A nomogram to predict the incidence of CR-POPF following OCP was constructed. The nomogram showed moderate predictive performance and may help identifying patients at high risk of CR-POPF and facilitating early individualized perioperative intervention.

## Data availability statement

The original contributions presented in the study are included in the article/supplementary material. Further inquiries can be directed to the corresponding authors.

## Author contributions

(I) Conception and design: LO, GJ and X-GH; (II) Administrative support: GJ, X-GH and GL; (III) Provision of study patients: GJ, X-GH, Y-JZ, GL, T-LH; (IV) Collection and assembly of data: LO, Y-WR, GN, R-DL; (V) Data analysis and interpretation: LO, R-DL, Y-WR, GN, Z-PH; (VI) Manuscript writing: LO; (VII) All authors contributed to the article and approved the submitted version.
